# The Aberdeen Hospital for Sick Children

**Published:** 1892-10-22

**Authors:** Katharine M. Lumsden

**Affiliations:** Hon. Superintendent, Hospital for Sick Children, and Secretary to the Aberdeen District Nursing Association


### HOSPITAL OPINION.

[Contributions critical, suggestive, controversial, and technical are invited for this column, which is open to everyone who has anything to say that is of practicalvalue and interest.]

### THE ABERDEEN HOSPITAL FOR SICK CHILDREN.

I see in your paper of October 8th a description of the Aberdeen hospitals. All connected with these institutions must heartily appreciate the friendly spirit in which it is written; and I should like, in particular, to acknowledge, on behalf of my sister, Miss Lumsden, of the Royal Infirmary, the well-deserved compliments paid to her work, both for this hospital and for the infirmary. It is, however, to be regretted that the—probably—short stay of your Commissioner in Aberdeen prevented his enquiring more thoroughly into the working of some other charities to which he refers. He has apparently been misinformed regarding the establishment of what he calls a "nurses' institute." A branch of Queen Victoria's Jubilee Nursing Institute has recently been formed in Aberdeen, and is actually working for the dispensary. The local committee of management which governs its affairs may be quite trusted to act in harmony with, and not "apart from," existing charities, and the recommendation that it should be worked "from the dispensary" has been given in apparent ignorance of the practice of the Jubilee Institute in other large towns, as well as in ignorance of the present arrangements in Aberdeen. The suggestion that a "nurses' home" should be established in connection with the Royal Infirmary seems rather vague. Is it meant that the "nurses' home" is to provide nurses for the paying or the non-paying classes? If for the last, the requirements of the poor are met by Queen Victoria's Institute, already in operation here. If for the first, your Commissioner seems to be also unaware of the fact that nurses for the paying classes can be supplied both from the infirmary and the Sick Children's Hospital. Owing to the hitherto insufficient accommodation at the infirmary, the largest share of this work has for the last few years been performed by this hospital, a great number of cases, both of adults and children, having been attended by its nurses. With the present improved arrangements, the infirmary will now, of course, be able to do its part. It is now the generally received opinion that nurses engaged in private work ought to be in touch with hospitals, and ought to have opportunities of doing regular duty in their wards. If a sufficiently large staff is maintained this can very well be done, to the advantage of the public, of the hospitals, and of the nurses themselves. Such is the practice of this hospital, and it is found satisfactory. But judging from my experience of the average annual demand for private nurses, and taking into consideration the low charges made in Aberdeen, and which it would not be advisable to raise to the London standard, I cannot think that the establishment of such a nurses' home, as may perhaps be intended in your Commissioner's report, would prove a paying, or even a self-supporting institution.

Your Commissioner would also propose to abolish our fever ward, one reason given being that the inhabitants of the neighbourhood would thus be freed from risk of infection. I cannot suppose that he shares the opinion held by some of the unenlightened public, that to look at the outside of a fever hospital is sufficient to give one scarlet fever; but I can assure him that the inhabitants of this neighbourhood run no risk whatever, and I must regret that I had not the opportunity of explaining to him the details of our management of the fever ward. I could also have informed him that the only fixed endowment belonging to this hospital is dependent on the possession of a ward for infectious diseases. The economy which he recommends would, therefore, cut two ways, while the loss of our fever ward would not only occasion us great trouble and inconvenience, but would deprive our nurses of some of their most valuable training.

As regards the commencement of this hospital, I know that Dr. Garden, who has rendered to it such valued service, would join me in regretting that no mention is made in your report of others who materially assisted in its establishment; and I would specially name Professor Stephenson and Sheriff Dove Wilson.

Katharine M. Lumsden,
Hon. Superintendent, Hospital for Sick Children,
and Secretary to the Aberdeen District Nursing Association.

[May we ask Miss Lumsden how long the branch of the Queen Victoria's Jubilee Nursing Institute has been formed in Aberdeen, and how many nurses are employed and paid by it at the present time? We believe that the movement is so recent as not even to have shaped itself definitely, and on the highest grounds of public policy any scheme of district nursing should be worked as a part of the dispensary if the best possible arrangement is desired by the citizens of Aberdeen. It is not the policy or the desire of the Jubilee Nursing Institute to interfere with the development of local institutions founded upon the best lines. Our proposal to establish a nurses' home in connection with the Royal Infirmary was certainly not vague, but definite. All that Miss Lumsden says goes to confirm the contention of our Commissioner, namely, that the establishment of such a nurses' home for the accommodation of a large body of probationer nurses would be an excellent scheme, having regard to the material for training which Aberdeen at present contains. A private nursing staff in number sufficient for local needs would necessarily be accommodated in the nurses' home, but the larger proportion of it should be taken up by probationers in training, many of whom would pay liberally for the advantages offered, and experience shows that under proper management financial success must follow its institution. Our ideal scheme includes the co-operation of all the institutions in Aberdeen, so that the probationers would have the fullest opportunity to secure an adequate training. As to the proposal to abolish the fever ward, which certainly should not be continued in its present position, is Miss Lumsden prepared to declare that the inhabitants in its neighbourhood incur no risk whatever from its present situation? If so, her views will not be supported by the teachings of experience in other places. Besides, it is wrong and unnecessary to have private fever hospitals, and the City Council should be called upon to make adequate provision for the work now done in the old chapel building, and to afford facilities for the training of nurses, all of which was embraced in the scheme propounded by our Commissioner. If Miss Lumsden will supply us with the names of "the others" who assisted materially in the establishment of the Children's Hospital, we shall be glad to publish them. To sum up, Miss Lumsden, we fear, regards the question at issue from too narrow and personal an aspect. The requirements of the sick of a great city should be looked at in the whole, and dealt with upon some scheme sufficiently comprehensive to secure the proper carrying out of the necessary work in the best possible way at the least possible cost. We, therefore, hope that public opinion in Aberdeen will support the views of our Commissioner, because it will then soon become an ideal city, so far as medical matters are concerned.—Ed. The Hospital.]

